# Multidrug-resistant Nontyphoidal *Salmonella* Hotspots as Targets for Vaccine Use in Management of Infections in Endemic Settings

**DOI:** 10.1093/cid/ciy898

**Published:** 2019-02-15

**Authors:** Samuel Kariuki, Cecilia Mbae, Robert Onsare, Susan M Kavai, Celestine Wairimu, Ronald Ngetich, Mohammad Ali, John Clemens, Gordon Dougan

**Affiliations:** 1 Centre for Microbiology Research, Kenya Medical Research Institute, Nairobi; 2 Bloomberg School of Public Health, Johns Hopkins University, Baltimore, Maryland; 3 International Centre for Diarrhoeal Disease Research, Bangladesh, Dhaka; 4 Department of Medicine, University of Cambridge, United Kingdom

**Keywords:** nontyphoidal *Salmonella*, hotspots, multidrug resistant, Kenya

## Abstract

**Background:**

*Salmonella* infections cause a disproportionately high number of deaths in Africa, especially among poor urban populations. The increasing level of multidrug-resistant (MDR) infections is a major cause of concern in these settings where alternative effective treatment is unavailable. Other options for management of these infections must be sought. The knowledge of hotspots in endemic settings can help to prioritize management and control measures in Kenya and the region.

**Methods:**

Using blood cultures, we investigated children presenting with fever of unknown origin for *Salmonella* infections. We performed antimicrobial susceptibility testing and whole genome sequencing to further characterize *Salmonella* isolates. Using Global Positioning System technologies, we mapped *Salmonella* isolates to households of patients in the study site and determined risk factors associated with high concentration of cases in particular sites.

**Results:**

A total of 281 *Salmonella* species (149 from blood and 132 from fecal samples) from febrile children <5 years of age were studied. These consisted of 85 *Salmonella* Typhimurium, 58 *Salmonella* Enteritidis, 32 other nontyphoidal *Salmonella* (NTS) serotypes, and 126 *Salmonella* Typhi. The prevalence of MDR invasive NTS (iNTS) was 77.2%, with 15% resistant to ceftriaxone, a drug that is last-line treatment for iNTS and other severe gram-negative infections in Kenya. Invasive NTS and *S*. Typhi together mapped around common water vending points and close to sewer convergence points in the highly populated village.

**Conclusions:**

These hotspots could be targeted for management and control strategies, including a combined introduction of typhoid and iNTS vaccines, aimed at reducing transmission in these endemic settings.

Nontyphoidal *Salmonella* (NTS) disease, a major cause of diarrheal disease globally, is estimated to cause 93 million enteric infections and 155 000 diarrheal deaths each year [1]. The Institute for Health Metrics and Evaluation estimated that enteric NTS disease was associated with 4 847 000 disability-adjusted life-years (DALYs) lost (70 DALYs /100 000 population) and 81 300 diarrheal deaths (1.2 deaths/100 000 population) in 2010 [2]. However, in sub-Saharan Africa (SSA), incidence of invasive NTS (iNTS) disease is even more widespread and severe (227 [range, 152–341] cases/100 000 population) and comprises the largest number of cases (1.9 [range, 1.3–2.9] million cases) [3, 4]. Among iNTS cases, it is estimated that 63.7% occurred in children <5 years of age globally, and 68.3% occurred in children <5 years of age in SSA [4]. The incidence of iNTS in a single site in Kenya in children aged <5 years was 36.6 per 100 000 person-years, being highest in infants aged <7 days (174/100 000 person-years) [5]. Risk factors for iNTS disease were human immunodeficiency virus (HIV) infection, malaria, and malnutrition; the case fatality ratio was 22.1% (71/321) in children aged <5 years and 36.7% (11/30) in adults. In cases that are not promptly treated, mortality can be >30% [6].

In SSA, multidrug-resistant (MDR) *Salmonella* serotype Typhimurium of a novel sequence type (ST) 313 has been reported from several countries including Kenya [7], Malawi [8], the Democratic Republic of the Congo [9], Nigeria [10], Ghana [11], South Africa [12], and Mozambique [13] where iNTS is endemic and produces septicemia in the absence of gastroenteritis. So far no animal reservoir has been identified, and it is hypothesized that transmission is human to human [14].

Increasing antimicrobial resistance in iNTS is of great global concern, and the situation is even more acute in low-income countries where empiric oral options for effective treatment of life-threatening invasive disease are being rapidly eroded. MDR iNTS has been reported in Kenya and Malawi [8, 15] and in other parts of SSA [16–18], posing a major challenge to treatment and management options. Where new effective antimicrobials are lacking, developments in vaccines offer hope for reducing the burden of iNTS in endemic settings in SSA.

Nairobi, Kenya’s largest urban center, has an estimated population of 4.5 million, a third of whom live in informal settlements that lack proper sanitary and clean water facilities. MDR enteric pathogens in these settings are a common cause of community-acquired infections [7, 19]. In the early 1990s, only 34.2% of iNTS from cases in urban sites were MDR (resistant to ≥3 antibiotics); the most common resistance phenotype was ampicillin, tetracycline, and co-trimoxazole in two-thirds of the isolates and chloramphenicol or ampicillin, tetracycline, and gentamicin in 15% of the isolates [7]. There were no significant differences in prevalence of resistance between the 2 major serotypes, *S*. Typhimurium and *Salmonella* Enteritidis. Ciprofloxacin and ceftriaxone were the only antibiotics to which all NTS isolates were fully susceptible. In subsequent studies in 2000–2010, the proportion of MDR isolates has risen to >75% [7]. Similarly, in Malawi [20], MDR *S*. Typhimurium accounts for 90% of all NTS bacteremia isolates from HIV-infected adults and from children <5 years of age with various causes of immunosuppression. In West Africa, MDR was common among iNTS cases, with decreased ciprofloxacin susceptibility and extended-spectrum β-lactamase production being reported in Ghana [11] and Burkina Faso [21]. In this review, we report an effort to map hotspots of invasive *Salmonella* disease in an informal settlement 20 km east of Nairobi city that can inform plans for targeted prevention strategies.

## METHODS

### Brief Description of the Study Site

**Figure 1.**
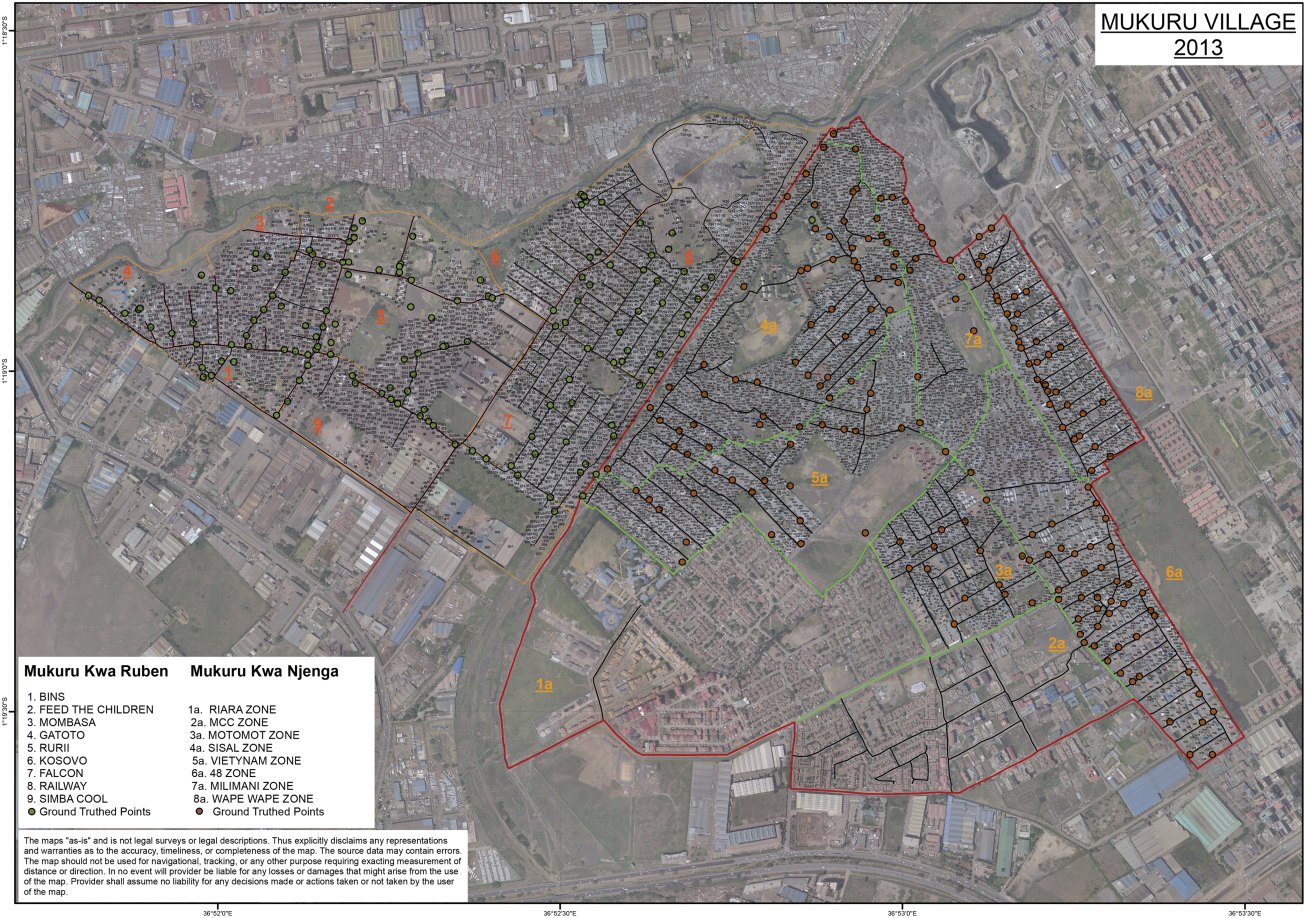
Demographic and disease surveillance site in the Mukuru informal settlement. The 2 villages where households were mapped (Mukuru kwa Ruben and Mukuru kwa Njenga) and a census was done are shown as darkened dots, and the numbers show the respective zones (zones 1–9 for Mukuru kwa Ruben and 1a to 8a for Mukuru kwa Njenga) for each village.

Our study site was Mukuru informal settlement, which is situated about 25 km east of Nairobi city center. It is one of the largest slums in the city with a population of around 250 000 people. The informal settlements are made up of improvised temporary houses often made from scrap materials, such as corrugated metal sheets, plywood, and polythene sheets. The settlements are densely populated and characterized by limited basic services and infrastructure for providing clean water, sanitation facilities, solid-waste management, roads, drainage, and electricity. In addition to poverty, a number of factors associated with informal settlements, including overcrowding, substandard housing, unclean and insufficient quantities of water, and inadequate sanitation, contribute to a high incidence of infectious diseases and increased mortality among those <5 years of age [22, 23]. Mukuru informal settlement is divided into 86 main villages: Mukuru Lunga-Lunga, Mukuru kwa Sinai, Mukuru kwa Reuben, Mukuru kwa Njenga, Mukuru Kayaba, and Mukuru North. The 2 major villages within the settlement, Mukuru kwa Njenga and Mukuru kwa Reuben with a combined population of 120 000, were mapped and a census was done. Mukuru kwa Reuben was demarcated into 9 zones with 7037 blocks, whereas Mukuru kwa Njenga had 8 zones with 8059 blocks (Figure 1). The area within the 2 villages studied consisted of mainly dwellings (98%) and a few godowns (ie, warehouses; 2% of the buildings) used by small-scale industrial set-ups in the outskirts of the villages. In the villages, a few goats and pigs owned by enterprising individuals roam the streets, eating from garbage dumps from vendors who sell vegetables along the village streets. In addition, the informal settlement in general has poor drainage, a common site of surface effluent throughout most of the mapped area. Three community health centers serve the population here: Missionaries of Mary, Mukuru Ruben, and Mukuru City Council. In total, 32 000 households were mapped.

### Study Subjects and Specimen Collection

The study subjects were patients aged 0–16 years who visited any of the 3 outpatient clinics in the study area seeking medical attention. The cases for iNTS investigation were defined as patients who had a subjective history of at least 3 days of fever and an axillary temperature of at least 37.5°C or who presented with a history of fever of any duration and had an axillary temperature of at least 37.5°C at presentation. Blood samples of 1–3 mL from children <5 years of age, and 5–10 mL from those aged 5–16 years, were taken and aseptically transferred into Bactec blood culture media (BD, Franklin Lakes, New Jersey). In addition, a fecal swab sample was taken from each patient using a sterile cotton-tipped swab and transferred into Cary-Blair transport media. The samples were then was transferred to the laboratory at the Centre for Microbiology Research, Kenya Medical Research Institute (KEMRI) within 4 hours. A structured questionnaire was used to elucidate the following information from each patient: clinical manifestations (eg, vomiting, fever, and/or dehydration), demographic data (age, sex, and residence), and types of stool samples (watery, mucous, bloody, or other form).

### Laboratory Procedures

#### Detection of Bacterial Pathogens

Blood cultures were incubated at 37°C in a computerized Bactec 9050 Blood Culture System (BD), and subcultured after 24 hours onto blood, chocolate, and MacConkey agar plates. The blood cultures were subsequently observed for a further 7 days for signs of bacterial growth (autodetection). A final subculture was performed for all blood cultures on the eighth day regardless of the state of bacterial growth. From the subcultures, bacterial isolates were identified using biochemical tests on API20E strips (API System, Montalieu Vercieu, France) and further typed by species-specific serological tests.

#### Fecal Cultures

The rectal swab or loopful of the stool specimen was transported to the KEMRI laboratory and initially cultured on selenite F (Oxoid, Basingstoke, United Kingdom) broth aerobically at 37°C overnight. Broth cultures were then subcultured on MacConkey agar and *Salmonella-Shigella* agar (Oxoid) and incubated at 37°C overnight. To identify suspected *Salmonella* bacteria, nonlactose-fermenting colonies were biochemically tested using triple sugar iron slants. From the subcultures, bacterial isolates were identified using biochemical tests on API20E strips and further typed by species-specific serological tests.

### Antibiotic Susceptibility Testing

Antibiotic susceptibility testing was performed using the disk diffusion technique for all commonly used antimicrobials in Kenya on Mueller-Hinton agar (Oxoid, Basingstoke, United Kingdom). For gram-negative enteric bacterial species (this includes ampicillin 10 µg, tetracycline 30 µg, gentamicin 10 µg, co-trimoxazole 25 µg, chloramphenicol 30 µg, co-amoxiclav 20:10 µg, cefuroxime 30 µg, ceftazidime 30 µg, ceftriaxone 30 µg, cefotaxime 30 µg, ciprofloxacin 5 µg, and nalidixic acid 10 µg), minimum inhibitory concentrations (MICs) were performed using E-test strips (AB BIODISK, Solna, Sweden). Results were interpreted according to the guidelines provided by the Clinical and Laboratory Standards Institute [24].

### Genetic Typing of Sequence Types and NTS Isolates

Genomic DNA from the NTS isolates was extracted using the Wizard Genomic DNA Extraction Kit (Promega, Fitchburg, Wisconsin). Two micrograms of genomic DNA was subjected to indexed whole genome sequencing (WGS) on an Illumina Hiseq 2000 platform at the Wellcome Trust Sanger Institute, to generate 100-bp paired-end reads. WGS was performed using new sequencing technologies (Illumina, 454) and exploited genotyping platforms (GoldenGate) for high-resolution and high-throughput sample analysis. The likelihood test ratios were determined as previously described [25]. The support for nodes on the trees was checked using 100 random bootstrap replicates. Resulting phylogenetic trees were visualized using the FigTree package version 1.4.0 (http://tree.bio.ed.ac.uk/software/figtree/). Subtrees were extracted for each subclade, which are therefore each rooted by the other subclades.

### Mapping Hotspots for NTS and Antimicrobial Resistance

Using the MicroReact software [26], the microbiological data on serotypes and genotyping data were entered into a model along with Global Positioning System (GPS) coordinates for each patient to map to potential spatial risk factors (eg, proximity to water or sewage sites, socioeconomic activities, health-seeking patterns).

### Ethical Considerations

The study was approved by the Scientific and Ethics Review Unit of KEMRI (Scientific Steering Commitee number 2076). All parents and/or guardians of participating children were informed of the study objectives, and voluntary written consent was obtained before inclusion. A copy of the signed consent was filed and stored in password-protected cabinets at KEMRI.

## RESULTS

### Study Population and Microbiological Investigation

Between January 2013 and December 2017, a total of 4150 blood cultures were processed from patients from the 3 outpatient clinics situated in Mukuru informal settlement (Figure 1), with 72% of those suspected to have fever with or without diarrhea being <5 years of age. From these, a total 147 of *Salmonella* species consisting of 50 (33.6%) *S.* Typhimurium, 34 (22.8%) *S.* Enteritidis, 48 (32.2%) *Salmonella* Typhi, and 17 (11.4%) other serotypes were isolated and further characterized using WGS.

From a total of 4055 fecal samples of the same patients (95 of whom could not give a fecal sample), 35 (26.5%) *S.* Typhimurium, 24 (18.2%) *S.* Enteritidis, 58 (43.9%) *S.* Typhi, and 15 other serotypes were isolated (Table 1). Of these, a total of 8 *S.* Typhimurium, 5 *S.* Enteritidis, and 22 *S.* Typhi came from patients whose blood cultures were also positive.

**Table 1.** Distribution of *Salmonella* Serotypes From Blood and Stool of Patents With Fever, Mukuru Informal Settlement, Kenya

Serotype	MMM	MR	MCC	Total, No. (%)
Blood	n = 1713	n = 1389	n = 1056	
*Salmonella* Typhimurium	23	18	9	50 (33.6)
*Salmonella* Enteritidis	11	12	11	34 (22.8)
Other NTS	2	5	10	17 (11.4)
*Salmonella* Typhi	25	13	10	48 (32.2)
				149
Stool	n = 1827	n = 1205	n = 1123	
*Salmonella* Typhimurium	16	14	5	35 (26.5)
*Salmonella* Enteritidis	10	9	5	24 (18.2)
Other NTS	7	5	3	15 (11.4)
*Salmonella* Typhi	20	21	17	58 (43.9)
				132

Data are presented as No. unless otherwise indicated.

Abbreviations: MCC, Mukuru City Council; MMM, Missionaries of Mary; MR, Mukuru Ruben; NTS, nontyphoidal *Salmonella*.

### Antimicrobial Susceptibility Testing

From a total of 101 iNTS isolates, 69 (68.3%) were resistant to at least one antimicrobial, and 58 (57.2%) were resistant to ≥2 commonly available antimicrobials. The most common resistance phenotypes were resistance to ampicillin, chloramphenicol, and co-trimoxazole. In addition, 15% of iNTS isolates were resistant to extended-spectrum β-lactams (cefotaxime or ceftriaxone), whereas 6% were resistant to ciprofloxacin.

### Mapping of Serotypes and Genotypes

**Figure 2.**
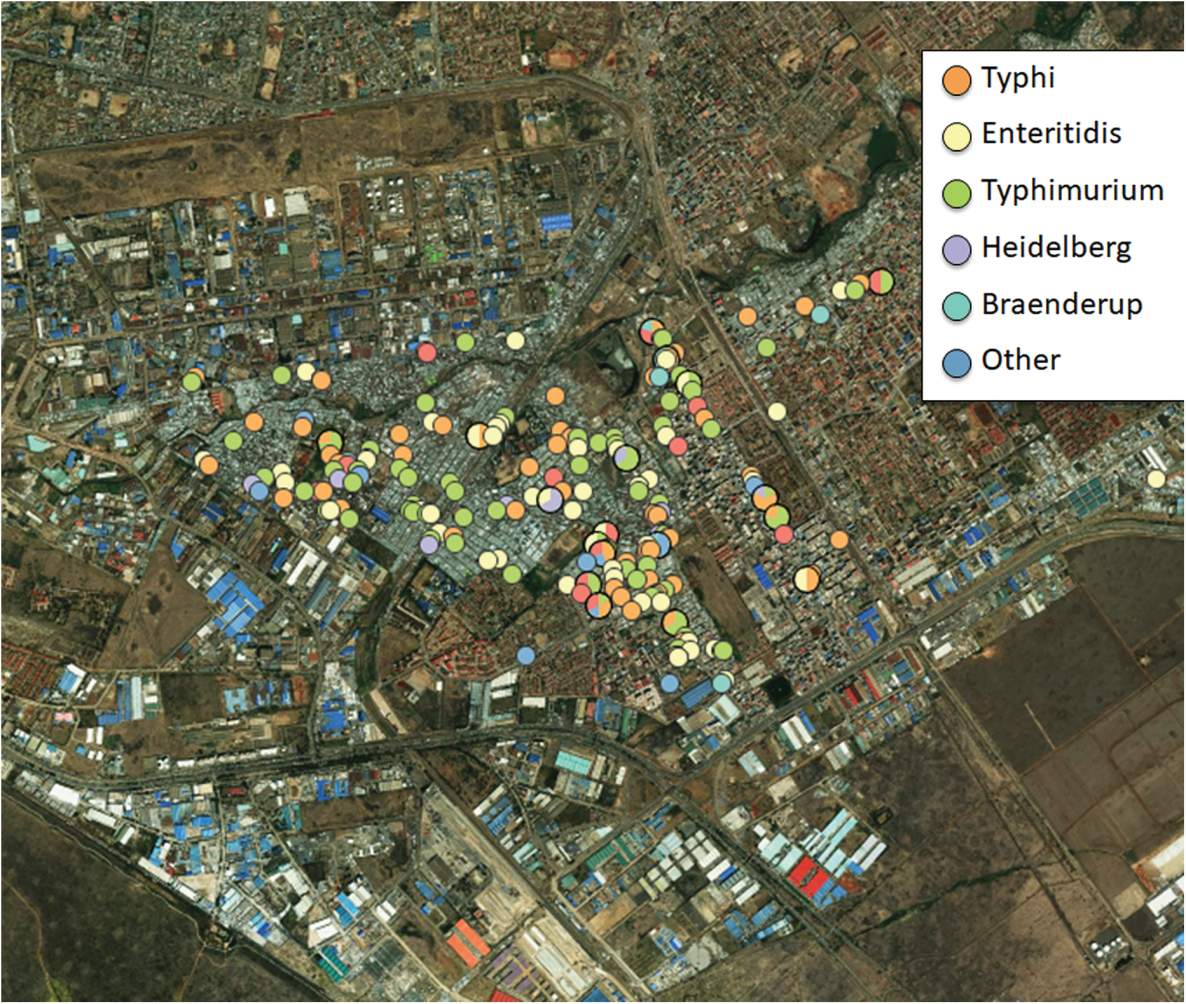
Hotspots of *Salmonella* serotypes in the 4 villages of Mukuru informal settlement. To the southeast of the map are godowns (warehouses) used by small-scale light industries.

Using GPS and proximity distance data, the iNTS and *S*. Typhi isolates appeared to be concentrated around the village, with the highest around water vending points, sewer convergence points, and shared toilet facilities (Figure 2). Indeed, the 2 infections iNTS and *S.* Typhi were closely related in geographic distribution in the mapped area of the study. From WGS, the main genotypes for *S.* Typhimurium were ST19 (33%) and ST313 (67%), whereas all *S.* Enteritidis isolates were ST11. Of the *S.* Typhi isolates sequenced, 96% were ST1, whereas 2% and 1% were ST2 and ST2230, respectively.

## DISCUSSION

Since 2000, the prevalence of multidrug resistance in invasive *Salmonella* disease especially for commonly available antimicrobials has been increasing steadily in Kenya [7, 15]. The increasing rate of antimicrobial resistance for iNTS is worrying as more effective antimicrobials are either unavailable or too expensive to be afforded by public healthcare facilities. Recently we documented the emergence of ceftriaxone-resistant invasive *S.* Typhimurium ST313 isolates in Kenya [25]. The spectrum of resistance in these isolates extends beyond the cephalosporins to include tetracyclines, co-trimoxazole, chloramphenicol, and aminoglycosides such as streptomycin. However, these isolates have remained susceptible to carbapenems and fluoroquinolones, but rising MICs for the latter antimicrobials are being observed in recent iNTS isolates. Illegal, commonly available antimicrobials are sold over the counter in these settings and indeed in many urban areas, and this would further fuel the rise of resistance. In Southeast Asia, fluoroquinolone resistance is now a growing problem in iNTS, with the first report of ciprofloxacin resistance in *Salmonella enterica* infection (eventually leading to treatment failure) being published in 1990 [27]. Alternative means for management of life-threatening iNTS will be required to forestall the high mortality rates associated with invasive disease in our informal settlement where disease is endemic.

In our study, typhoid affected most of the older children (72% were >5 years of age), whereas iNTS affected mainly younger children (78% were <5 years of age) and symptoms were often more severe compared with those of typhoid fever. In studies in rural and urban informal settlements in Kenya, the local minimum incidence of community-acquired iNTS was estimated at 166 per 100 000 per year for children <5 years of age [5, 28]. The adjusted incidence in the rural site was 568/100 000 person-years, and that in the urban site was 51/100 000 person-years. The incidence was highest in children <5 years old with mortality up to 28%. However, rates in both sites were thought to be underestimated by 4- to 8-fold due to incomplete blood culturing of febrile patients and use of healthcare facilities other than those used for the study [28], whereas typhoid disease was more important among the children in poor informal urban settings in Kibera, very similar to our study site in Mukuru informal settlement. These 2 informal settlements together account for nearly 800 000 population. However, our study observed almost equally high prevalence of both iNTS and typhoid in the same settings of the poor informal settlement. This would call for a common effort to control both of these endemic infections.

The STs most associated with iNTS disease are *S.* Typhimurium ST313 (67%) and *S.* Enteritidis ST11 (100%). Previous studies have shown that ST313 has been increasing in incidence across many SSA countries and now dominates most invasive *Salmonella* disease occurring in children <5 years of age [8, 29, 30]. In comparison, *S.* Enteritidis and *S.* Typhi manifest as a milder disease, with typhoid occurring more in older (aged 5–16 years) children.

As more people migrate into cities looking for jobs, the informal settlements are likely to grow and, unfortunately, the infrastructure, including sanitation, sewer disposal, and provision of clean drinking water, does not keep pace with this population growth. It will be important to consider short- to medium-term measures to prevent and control endemic infections including iNTS in this vulnerable population. There are already well-studied and World Health Organization (WHO)–recommended vaccines for typhoid [31]; these should be rolled out immediately in these settings. The mapping of hotspots in these endemic settings where 250 000 people live in densely populated structures would be an ideal starting point for such an iNTS prevention strategy. In addition, as observed in the hotspot map, iNTS and typhoid appear to be closely related in their dispersion in the 2 villages. It is likely that the 2 infections have similar routes of transmission. Previously we showed that iNTS disease in Kenya and the region is likely to be transmitted person to person [14], very similar to typhoid. Previous studies have also shown that local strains of *S.* Typhimurium ST313 [32] and *S.* Enteritidis ST11 [33] have gradually shed genes in a similar manner to *S.* Typhi evolution toward becoming human-adapted pathogens. Hence, methods for intervention to control one disease will easily lend to control and management of the other. As efforts toward developing vaccines against the most common and severe disease causing serotype iNTS serotype are under way, it would be prudent to consider deployment of vaccines initially in the mapped hotspots of these infections in the endemic sites. 

In conclusion, we have mapped hotspots of iNTS in an endemic setting for both typhoid and NTS disease. The increasing prevalence of multidrug resistance among iNTS strains is another compelling reason to consider these hotspots for management through vaccination programs that could also include WHO-recommended typhoid vaccines.

## Notes

***Author contributions.*** S. K.: manuscript preparation and field/laboratory data collection. C. M.: field epidemiology. R. O., S. M. K., R. N., C. W.: laboratory data analysis. M. A., J. C., G. D.: data analysis, manuscript preparation.

***Acknowledgments.*** The authors thank the director of KEMRI for support in publishing these data, as well as the field and laboratory staff of the Mukuru kwa Njenga Invasive *Salmonella* Disease Surveillance Project.

***Supplement sponsorship.*** This supplement was sponsored by Coalition against Typhoid, Sabin Vaccine Institute, Bill & Melinda Gates Foundation.

***Potential conflicts of interest.*** All authors: No reported conflicts of interest. All authors have submitted the ICMJE Form for Disclosure of Potential Conflicts of Interest. Conflicts that the editors consider relevant to the content of the manuscript have been disclosed.
